# Meetings of Societies

**Published:** 1892-12

**Authors:** 


### MEETINGS OF SOCIETIES.

### Bristol Medico-Chirurgical Society.

*Annual Meeting, October 12th,* 1892.

Mr. F. R. Cross, President, in the Chair.

After a vote of thanks to the retiring President (Mr. F. R. Cross) had been unanimously passed, his successor, Dr. E. Markham Skerritt, took the Chair, and read his inaugural address (see page 229), for which a cordial vote of thanks was given him. The Honorary Secretary, Assistant-Editor, and Honorary Librarian presented their annual reports, which were adopted. The following officers were chosen for the ensuing year:—President-Elect; Mr.

Greig Smith: Honorary Secretary; Mr. Munro Smith: Honorary Librarian; Mr. L. M. Griffiths: Members of Committee; Dr. Michell Clarke, Mr. J. Dacre, Dr. A. J. Harrison, Mr. W. H. Harsant, Mr. A. W. Prichard, Dr. J. E. Shaw.

Dr. George Parker showed: (1) Two cases of congenital de formity of the forearms in children. Both hands were fixed in the position of pronation. In one case the two radii were subluxated backwards. (2) A patient with a small branchial fistula between thyro-hyoid and sub-hyoid arches on left side. Other members of the same family showed signs of this defect. (3) A case of elephantiasis of the right leg. The patient had suffered from attacks of deep dermatitis. The skin was generally smooth, with occasional cracks and with some nodular growths beneath.

---

*November 9th*, 1892.

Dr. E. Markham Skerritt, President, in the Chair.

The proposal for the amalgamation of the Libraries of the Society and of the Medical School, which had already been approved by the Faculty of the School and by the Committee of the Society, was brought before the meeting by the President. After some discussion, the scheme was adopted.

It was proposed that Law 7 shall in future read as follows: "The subscription to the Society shall be one guinea, payable in advance." This was carried.

Dr. Michell Clarke showed a boy with well-marked Addison's disease, and two patients (boys) with Friedreich's hereditary ataxy. He pointed out the resemblances and differences between this condition and chorea and ordinary ataxy, such as the early age of the patient, the affection of more than one member of a family, absence of knee-jerk, slight nystagmus, &c.

---

*December 14th*, 1892.

Dr. E. Markham Skerritt, President, in the Chair.

The alteration in Law 7, relative to the increase of subscription from 10/6 to one guinea, was confirmed.

A modification of the Library Scheme (Rule I.) was adopted.

Mr. F. R. Cross, Mr. L. M. Griffiths, and Dr. Shaw, were elected to represent the Society on the conjoint Library Committee.

Dr. Aust Lawrence read notes on a case of extra-uterine gestation. The patient showed signs of pregnancy, and seven weeks after her last menstrual period was attacked with sudden, sharp abdominal pain. She had several subsequent attacks, and a few weeks later had a very severe one with collapse. The abdomen was opened, the ruptured tube found, and the cavity cleaned and drained. The woman made a good recovery.—Mr. C. A. Morton explained the pathological condition by a diagram, and exhibited the specimen. At the first attack of pain the distended Fallopian tube had apparently broken into the broad ligament, and later on the sac so formed had ruptured into the peritoneal cavity.

Mr. Greig Smith showed a number of specimens removed by operation, including the following:—Abortive extra-uterine fœtation in the broad ligament; inflamed and adherent uterine appendages; cancerous tumour of the umbilicus; tumour of a patent Meckel's diverticulum, formed chiefly of hypertrophied mucous membrane; cancerous growth from man's ear; concretion in vermiform appendix; adenomatous tumour of breast of aged woman; large myoma with nodules on surface of uterus; another large and soft myoma; several ovarian tumours; papillary carcinoma of breast; and scirrhus from the breast of a woman aged 27, with secondary nodules. The tumour had grown rapidly since the birth of a child.

Dr. Rogers read a paper on the relation between megrim and epilepsy. He remarked on the tendency of these diseases to be hereditary; and the occasional disappearance of one to give place to the other in the same individual. He narrated the case of a lady who had suffered severely from sick headaches, which ceased at the age of fifty, when she suffered from marked epileptic attacks. Both diseases might be considered paroxysmal neuroses. He commented on the amnesia sometimes present in megrim, and on other motor disturbances.

G. Munro Smith, *Hon. Sec.*

---

### Plymouth Medical Society.

*October 29th*, 1892.

Mr. T. Leah, President, in the Chair.

Dr. Lawrence Fox read a paper on empyema, in which, after sketching the views held by Hippocrates, Pinet, and Laennec, he remarked on the much earlier treatment of our cases than formerly obtained, and the importance, especially in children, of accurate diagnosis. He cited cases of children even with large collections of pus, and yet normal temperatures. He urged the value of the cyrtometer; but admitted that in children the diagnosis had often to be made by the hollow needle, a means not frequently enough employed. After dwelling on the differential diagnosis, Dr. Fox went on to discuss the treatment by (1) aspiration, (2) incision, and (3) incision with resection of rib. He said the first method was almost entirely superseded by (2) and (3), although aspiration by bringing negative pressure to bear helped expansion of the lung; it, especially in private practice, offered a happy medium between "trusting to nature" and having "a surgical operation performed." He recommended the ninth interspace between the mid- and post-axillary lines as the most suitable incision; but condemned irrigation except n septic cases, and considered sixteen days to four months as the usual time required for retaining the drainage tube. In his opinion, resection of a rib was an unnecessary procedure in most cases, and he recommended chloroform warily given as the best anæsthetic. When the sinus was closed, calisthenic exercise and forcible expiratory efforts with closed glottis were recommended as aids to expansion of the lung. Not long ago retraction of the chest, spinal curvature, discharging sinuses, amyloid disease, were often met with as relics of a bygone empyema, and we must congratulate ourselves on the much better results now obtained.—Mr. Lucy asked Dr. Fox if he had met with hemorrhoids accompanying empyema in children, and cited a case. He also recommended the injection of glycerine and water when using the exploring syringe, and asked Dr. Fox if he had met with any sudden deaths of patients suffering from empyema.—Mr. A. C. Rendle recommended an incision below the scapular angle.—Mr. Woollcombe alluded to the differentiation between pus in pleura and that between liver and diaphragm, and asked Dr. Fox as to the frequency of cerebral abscess (Godlee). He considered sudden death during irrigation due to the bursting of adhesions and shock.—Mr. Leah cited a case, in which during irrigation the patient complained of the taste of carbolic acid, and became worse after each washing out.—Mr. B. Rendle related a case of recovery in a boy after four aspirations.—Mr. F. Williams suggested the use of drainage tubes, stiffened with an inner wire spiral. —Dr. Fox, in replying, thanked Mr. Lucy for the suggestion of using fluid in the exploratory syringe, and considered that cerebral abscess was due to emboli passing the lung capillaries. He gave the case of a lunatic, whose pet delusion was that his chest was full of corruption, which proved after death to be only too true.

Reginald H. Lucy, *Hon. Sec.*

